# Clinical and biochemical characterization of hereditary transthyretin amyloidosis caused by E61K mutation

**DOI:** 10.3389/fnmol.2022.1003303

**Published:** 2022-10-12

**Authors:** Xujun Chu, Mengdie Wang, Ran Tang, Yanan Huang, Jiaxi Yu, Yunfeng Cao, Yilei Zheng, Zhiying Xie, Jianwen Deng, Zhi Wang, Wei Ma, Wenjing Song, Yuan Wu, He Lv, Wei Zhang, Zhaoxia Wang, Yun Yuan, Yu Liu, Lingchao Meng

**Affiliations:** ^1^Department of Neurology, Peking University First Hospital, Beijing, China; ^2^CAS Key Laboratory of Separation Science for Analytical Chemistry, Dalian Institute of Chemical Physics, Chinese Academy of Science, Dalian, China; ^3^University of Chinese Academy of Sciences, Beijing, China; ^4^Dong’e County People’s Hospital, Liaocheng, China; ^5^Shanghai Institute for Biomedical and Pharmaceutical Technologies, NHC Key Laboratory of Reproduction Regulation, Shanghai Engineering Research Center of Reproductive Health Drug and Devices, Shanghai, China; ^6^Department of Neurology, The First Affiliated Hospital of Nanchang University, Nanchang, China; ^7^Department of Cardiology, Peking University First Hospital, Beijing, China; ^8^Department of Ophthalmology, Peking University First Hospital, Beijing, China

**Keywords:** E61K mutation, kinetic stability, thermodynamic stability, TTR tetramer, kinetic stabilizers, ATTRv amyloidosis

## Abstract

**Objects:** This study was intended to find out more about the clinical characterizations of patients carrying transthyretin (TTR) E61K (p.Glu81Lys) gene mutation and the biochemical characterization of this mutant protein.

**Materials and methods:** Five patients who had been diagnosed with hereditary transthyretin amyloidosis and two asymptomatic carriers carrying TTR E61K gene mutation were reported. Biochemical and biophysical tests were conducted to observe the thermodynamic and kinetic stability. Fibril formation tests measured by turbidity assay were performed to explore the pathogenicity of this mutation. Kinetic stabilizer responsiveness was measured to determine the inhibitory effect on protein aggregation.

**Results:** The average age of onset for the five patients was 62 years, and the course of the disease ranged from 2 to 10 years. Cardiac disease was prominent in this group of patients. Nerve pathology revealed a mildly to moderately reduced myelinated fiber density and muscle pathology showed predominant neurogenic impairment accompanied by possible myogenic impairment. E61K-TTR was characterized as a kinetically destabilized protein compared to WT-TTR but its thermodynamic stability was not compromised. In addition, the subunit exchange of E61K with WT-TTR further destabilized the heterozygous tetramer. Meanwhile, the E61K:WT heterozygous tetramer exhibited a poor response to kinetic stabilizers in the fibril formation assay. Finally, the serum TTR tetramer concentration was low in E61K-TTR symptomatic patients and in one asymptomatic gene carrier. Vyndamax (Tafamidis) could increase the TTR tetramer concentration.

**Conclusions:** Patients with E61K mutation tended to be late-onset. The concentration of TTR tetramer in the serum might serve as a biomarker to monitor disease progress, therapeutic window time, and therapeutic response to TTR kinetic stabilizer drugs.

## Introduction

Prealbumin, commonly known as transthyretin (TTR), is a tetrameric protein consisting of 127 amino acids with a molecular weight of 55 kDa (Kanda et al., 1974). It is primarily synthesized and secreted by the liver (Soprano et al., 1985), but also produced in small amounts by the retinal pigment epithelium and the choroid plexus of the brain (Stauder et al., 1986) and also synthesized in the pancreas, Schwann cells, and neurons under stress (Refai et al., 2005; Murakami et al., 2010) before being secreted into the blood or cerebrospinal fluid, performing such functions as transporting thyroid hormones and binding retinol binding protein (RBP; Raz and Goodman, 1969; Monaco et al., 1995). Normally, tetrameric TTR consists of four homozygous monomers. In hereditary transthyretin amyloidosis (ATTRv amyloidosis), genetic mutations on TTR proteins usually compromise their stability, causing TTR dissociation into dimers and monomers. The dissociated TTR monomer quickly misfolds into partially unfolded conformation, self-assembles into toxic non-fibrillar aggregates, and later becomes amyloidogenic fibers (Hammarström et al., 2002). These amyloid proteins are deposited in tissues such as the peripheral nervous system and the myocardium (Koike et al., 2004), leading to hereditary transthyretin amyloid polyneuropathy (ATTRv-PN) and hereditary transthyretin amyloid cardiomyopathy (ATTRv-CM).

ATTRv-PN mainly affects peripheral and autonomic nervous systems. Sensorimotor polyneuropathy, autonomic dysfunction, and gastrointestinal disturbances are the main clinical manifestations of the disorder, which may lead to death after 7.3 years of the disease onset (Conceição and De Carvalho, 2007; Koike et al., 2012). For ATTRv-CM, amyloidogenic fibers can infiltrate all cardiovascular structures, including the conduction system, atrial and ventricular myocardium, valvular tissue, and coronary and aortic arteries (Wang et al., 2022), with myocardial infiltration leading to a progressive increase in the thickness of the right and left ventricular walls, ultimately leading to heart failure (Buxbaum et al., 2006).

So far, more than 130 destabilized mutations on the TTR gene have been reported, most of which can lead to TTR amyloidosis. ATTRv amyloidosis often impacts the quality of life, independence, and life expectancy of patients. Multiple lines of clinical evidence have suggested that ATTRv amyloidosis patients carrying TTR mutations may have vastly different clinical manifestations, disease age of onset, progressiveness, mutant penetrance, and drug response, posing clinical challenges to precise diagnosis and treatment options. Biochemical and biophysical characterizations of mutations can partially account for these clinical observations and assist clinicians in providing precision health care for ATTRv amyloidosis patients.

In our center, 60 unrelated families with ATTRv amyloidosis have been identified by sequencing the TTR gene. Currently, V30M (p.Val50Met) and A97S (p.Ala117Ser) are the most common genotypes in our cohort, both of which have been reported more often. The third common genotype is E61K (p.Glu81Lys). Only a small number of cases with E61K mutation have been reported, mainly in Japan (Shiomi et al., 1993; Noto et al., 2009; Murakami et al., 2017, 2021; Nakano et al., 2018). However, its biochemical properties were not systematically characterized. In this study, five ATTRv amyloidosis families carrying TTR E61Kmutation and their clinical symptoms were reported. More importantly, the biochemical properties, plasma concentration, and drug response of E61K mutant TTR were characterized to gain mechanistic insights into this disease mutation.

## Material and Methods

### Clinical assessment

Five patients and two asymptomatic carriers from five unrelated families, who had been diagnosed with ATTRv amyloidosis complicated with TTR E61K mutation at the Department of Neurology, Peking University First Hospital, between April 2015 and October 2021, were included in this study. All patients were inquired about their disease history and had focused neurological examination and measurement scales performed, including the progressive course of the disease and Coutinho stages (stage 0, no symptoms; stage I, unimpaired ambulation, mostly with mild sensory, motor, and autonomic neuropathy in the lower limbs; stage II, assistance for ambulation required, mostly with a moderate motor, sensory, and autonomic impairment of the four limbs; stage III, wheelchair-bound or bedridden status with severe sensory, motor, and autonomic involvement of all limbs). Cardiac parameters of echocardiography and 99mTechnetium-pyrophosphate (99mTc-PYP) scintigraphy results were collected. All patients performed Neuropathy Impairment Score (NIS), Norfolk Quality of Life-Diabetic Neuropathy (Norfolk QOL-DN) score, and Composite Autonomic Symptom Score 31 (COMPASS-31). All patients and asymptomatic carriers provided their informed consent for this study, and ethical approval was obtained from the Human Research Ethics Committee of Peking University First Hospital.

### Electrophysiological assessment

All patients were subjected to peripheral nerve conduction studies (Alpine Biomed ApS corporation in Denmark). The compound muscle action potentials (CMAPs), distal motor latencies (DMLs), and motor conduction velocities (MCVs) were recorded for the median nerves, ulnar nerves, tibial nerves, and peroneal nerves with surface electrodes. The sensory nerve action potentials (SNAPs) and sensory conduction velocities (SCVs) were recorded with surface electrodes from the median nerves, ulnar nerves, superficial peroneal nerves, and sural nerves.

### Pathology

A combined biopsy of the sural nerve and gastrocnemius was performed in patients 1 and 2. One specimen of the sural nerve was fixed in 4% formaldehyde, paraffin-embedded, 8-μm sections, and stained with hematoxylin and eosin (HE), Congo-red. TTR immunohistochemical staining was also performed by standard techniques (DAKO). Another specimen was fixed in 3% glutaraldehyde, postfixed in 1% osmium tetroxide, dehydrated through acetone, and embedded in Epon 812. Semithin sections for light microscopy were stained with toluidine blue. The muscle specimens were frozen in isopentane and cooled in liquid nitrogen. Serial frozen sections were stained, using routine histological and enzyme histochemical methods, and immunohistochemical techniques for TTR (DAKO).

### Plasmid construction and protein purification

Wild-type (WT) TTR and variants were recombinantly prepared following previous literature (Nakano et al., 2018). Genes of *E. coli* transthyretin were codon optimized, synthesized by GenScript, and sub-cloned into pET-29b (+) vectors. WT, L55P (p.Leu75Pro; E61K plasmids were transformed into BL21 (DE3) *E. coli* cells respectively. Cells were grown to OD600 at 0.6–0.8 before being induced by IPTG (0.5 mM) at 37°C for 4 h. Cultured cells were harvested and resuspended in resuspension buffer (50 mM Tris, 150 mM NaCl, pH = 7.5; 15 ml buffer/L of culture). Cells expressing recombinant proteins were thawed and lysed by sonication at 4°C. Lysed cells were centrifuged for 30 min at 16,000 rpm. Ammonium sulfate (final concentration of 242 g/L) was slowly added to the supernatant with vigorous stirring at 4°C for 20 min. The solution was centrifuged at 12,000 rpm for 15 min at 4°C. The supernatant was supplemented with additional ammonium sulfate to a final concentration of 607 g/L with vigorous stirring at 4°C for 20 min. The solution was centrifuged at 12,000 rpm for 15 min at 4°C. The pellet was resuspended in 20 ml of anion exchange buffer A (25 mM Tris, 1 mM EDTA, pH = 8.0) and dialyzed against 4 L of buffer A overnight at 4°C. After dialysis, the sample was filtered and applied to a 50 ml Source 15Q anion exchange column (GE Healthcare) equilibrated with buffer A. TTR was eluted using a linear gradient of NaCl (160 ml; 50–350 mM) followed by a NaCl wash (50 ml; 350 mM). Eluted TTR was purified using a 120 ml Superdex 200 gel filtration column (GE Healthcare) in SEC buffer (10 mM sodium phosphate, 100 mM KCl, 1 mM EDTA, pH = 7.4). The protein containing fractions were identified by SDS-PAGE gel analysis, pooled, and concentrated. No significant impurities were identified and purity was estimated to be 98% based on SDS-PAGE electrophoresis analysis.

### Thermodynamic stability of TTR was measured by urea-mediated tryptophan fluorescence

The thermodynamic stability of the three proteins WT-TTR, L55P-TTR, and E61K-TTR (3.6 μM) was measured at different concentrations of urea between 0.5 and 9 M in phosphate buffer (10 mM sodium phosphate, 100 mM potassium chloride, 1 mM EDTA, pH = 7.4). Excitation at 295 nm was used and the fluorescence emission intensity of tryptophan from 305 to 405 nm was collected. The ratio of 355 to 335 nm fluorescence intensity reflected the degree of TTR dissociation.

### Kinetics of monomer unfolding and tetramer dissociation as a function of urea

The monomer unfolding is much faster than the dissociation of the tetramer, so the rate-limiting of TTR dissociation is the structural changes from quaternary to tertiary, which is measured by tryptophan fluorescence (I355/I335). The rates of tetramer dissociation were determined using TTR (3.6 μM) in 3–9 M urea in phosphate buffer (10 mM sodium phosphate, 100 mM KCl, 1 mM EDTA, pH = 7.4, 25°C) as a function of time. The kinetics data fit well to a single exponential function: I355/335 = I355/335N + A (1 − e-kdiss t); where I355/335N is the native protein fluorescence intensity ratio (355/335 nm), A is the amplitude difference, kdiss is the tetramer dissociation rate constant and t is time in hour. The lnkdiss vs. urea concentration plot is linear, allowing extrapolation to 0 M urea.

### Fibril formation assay

TTR (7.2 μM) was incubated with different concentrations of Tafamidis, Diflunisal, and AG10 in phosphate buffer (10 mM sodium phosphate, 100 mM potassium chloride, 1 mM EDTA, pH = 7.4) for 0.5 h at 37°C before acidic buffer (NaOAc 200 mM, KCl 100 mM, acidified by AcOH to pH = 4.4) was added in equal volume to allow for amyloid formation. The degree of TTR amyloid formation was measured by OD330 after all samples were incubated at 37°C for 72 h.

### Analysis of tetramer TTR concentration in patients’ serum samples by Ultra-Performance Liquid Chromatography (UPLC)

UPLC (FLR) Waters Acquity H-Class plus pro and Protein-pakTM Hi RCSQ (5 μm, 4.6 × 100 mm) were from Waters. The A2 molecule was synthesized in the laboratory as previously reported. Plasma from six E61K mutation carriers was filtered through 0.22 μm PVDF membrane, followed by 5 μl of each filtrate, 5 μl of PBS was added, mixed well and 1 μl of A2 (1 mM) was added. After 2 h of incubation at 25°C, the mobile phase was added at a starting rate of 45 μl, vortexed, and mixed thoroughly, and the supernatant was centrifuged (15,000 *g*, 4°C, 5 min) for sample analysis. Each sample was loaded in a volume of 20 μl into a Waters Acquity H-Class UPLC plus pro instrument with a Waters Protein-pakTM Hi RCSQ (5 μm, 4.6 × 100 mm). TTR was eluted from the column in a gradient using buffer A (25 mM Tris-HCl, pH = 9.0, 1 mM EDTA) and buffer B (1 M NaCl, 1 mM EDTA, 25 mM Tris-HCl, pH = 9.0) at a flow rate of 0.6 ml/min.

### TTR subunit exchange reaction and hetero-tetramers formation

WT-TTR and E61K-TTR were mixed in equal amounts in phosphate buffer (10 mM sodium phosphate, 100 mM KCl, 1 mM EDTA, pH = 7.4) at 3.6 μM and incubated at 4°C for 48 h for sufficient subunit exchange reaction as previously reported (Robinson and Reixach, 2014). During this process, WT-TTR and E61K-TTR tetrameric proteins dissociate into monomers and reassemble to form heterozygous tetramer proteins containing five different and exchangeable monomeric components (4WT-TTR, 3WT•1E61K-TTR, 2WT•2E61K-TTR, 1WT•3E61K-TTR, 4E61K-TTR). However, these five components are not further separable as they constantly undergo subunit exchange and stay in exchangeable equilibrium. Subsequently, the mixture of heterozygous proteins obtained after subunit exchange was tested for their kinetic and thermodynamic stability as well as to measure the small molecule stabilizing effect.

## Results

### Clinical features

**Figure 1:**
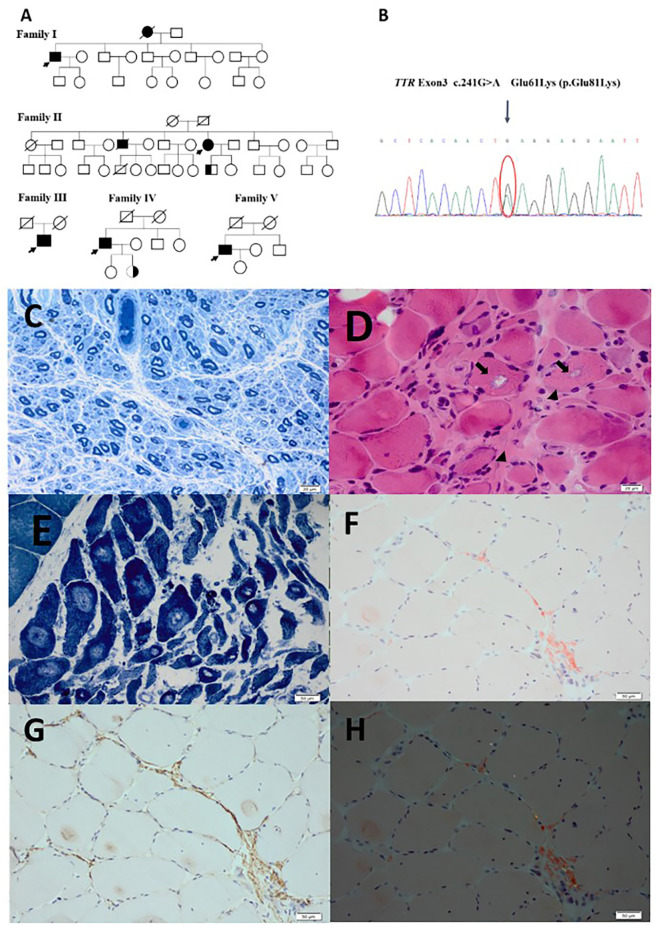
**(A)** Genograms of five patients and two asymptomatic carriers. **(B)** The sanger sequencing of patient with E61K mutation on TTR gene. **(C)** Severely reduced myelinated fiber densities on the semithin section in patient 2. **(D)** Atrophic angular and round muscle (arrow heads) fibers and some muscle fibers with rimmed vacuoles (arrows) in patient 1. **(E)** Typical target-like muscle fibers with nicotinamide adenine dinucleotide tetrazolium reductase staining were found in patient 2. **(F)** Amyloid deposits in perimysium and perivascular areas on Congo-red staining in patient 2. **(G)** Amyloid deposits in perimysium and perivascular areas on TTR immunohistochemical staining in patient 2. **(H)** Apple-green birefringence viewed under polarized light in patient 2. Scale bar = 20 μm in **(C,D)**. Scale bar = 1 μm in **(E,F,G,H)**.

All five patients and two asymptomatic carriers were of Chinese Han ethnicity, including five males and two females, with pathogenic variation of TTR c.241G > A, p.Glu61Lys (Figures 1A,B). All five patients were late-onset and the mean age at onset was 62 years (ranging from 59 to 63 years). The mean course of the disease was 4.8 years (ranging from 2 to 10 years). Patient 1, 2, and 4 had a positive family history. For the initial symptoms, three patients presented with painful paraesthesia, one presented with alternation of diarrhea and constipation, and one had weakness in the lower limbs. All the patients had sensory-motor polyneuropathy and autonomic nerve system involvement, including varying degrees of diarrhea and constipation, sexual dysfunction, urination abnormality, postural dizziness, abnormal sweating, xerostomia, and skin color changes (Table 1). Autonomic symptoms were more severe for patients 1, 2, and 4 than for others, with higher COMPASS-31 than those of others. Three patients developed cardiac symptoms, including shortness of breath after exercise for patients 2, 4, and 5, and palpitation for patient 2. Patient 1 had mildly elevated NT-proBNP and patient 2 had significantly elevated NT-proBNP with atrial fibrillation. All patients were examined by echocardiography, showing thickened interventricular septal and left ventricular posterior wall. Patient 1, 2, and 4 underwent 99mTc-PYP scintigraphy, revealing increased PYP uptake in the heart with a visual score of grade 3 (Table 2). All five patients underwent varying degrees of body weight loss, and patient 4 suffered from chronic cough. Patient 4 took Vyndamax (Tafamidis) 61 mg orally once daily. Patient 5 took diflunisal orally daily and stopped it 6 months ago. The rest of the patients received no disease-modifying therapies. The two asymptomatic carriers were patient 2’s 40-year-old son and patient 4’s 35-year-old daughter, both of whom did not present any symptoms currently.

**Table 1:** Characteristics of symptomatic Glu61Lys-related ATTRv patients in this study and review.

**Patient**	**Sex**	**AO (years)**	**Course of disease (years)**	**Family history**	**Initial symptoms**	**Peripheral neuropathy**	**Autonomic neuropathy**	**Cardiac disease**	**Other abnormalities**	**Reference**
1	M	60	4	Positive	Lower limbs weakness	SMPN for 4 years	ADC, dysuria, xerostomia, erectile dysfunction, skin color changes, abnormal sweating	Cardiac hypertrophy	BW loss	This study
2	F	62	2	Positive	Painful paraesthesia	SMPN for 1.5 years	Constipation, hiccup, frequent urination	Atrial fibrillation, Cardiac hypertrophy	BW loss	This study
3	M	64	10	Negative	ADC	SMPN for 0.5 years	ADC	Dilated cardiomyopathy	BW loss	This study
4	M	59	4	Positive	Painful paraesthesia	SMPN for 4 years	Diarrhea, abnormal sweating, OH, dysuria	Cardiac hypertrophy	BW loss, cough	This study
5	M	63	4	Negative	Painful paraesthesia	SMPN for 4 years	ADC, abnormal sweating, frequent urination	Cardiac hypertrophy, pacemaker implantation	BW loss	This study
Family 1	F	63	13	Positive	Numbness	SMPN for 6 years	Diarrhea, OH	Cardiac hypertrophy, restrictive diastolic dysfunction, pacemaker implantation	NA	Murakami et al. (2017)
Family 2	M	62	2	Positive	Diarrhea	SMPN for 2 years	Diarrhea	NA	NA	Shiomi et al. (1993)
Family 3	M	55	11	Positive	Syncope	SMPN for 0.5 years	OH, urinary retention and impotence	Cardiac hypertrophy, diastolic dysfunction, PVC, PAC	NA	Noto et al. (2009)
	F	65	3	Positive	CTS	NA	NA	Cardiac hypertrophy, congestive heart failure,	NA	Noto et al. (2009)
Family 4	M	77	NA	Negative	Pretibial edema	SMPN	Constipation, OH	NA	NA	Nakano et al. (2018)
Family 5	F	67	NA	NA	pretibial edema, dyspnea	SMPN for 3 years	NA	NA	NA	Nakano et al. (2018)

*Abbreviations: ADC, alternation of diarrhea and constipation; AO, age of onset; BW, body weight; CTS, carpal tunnel syndrome; F, female; M, male; NA, not available; SMPN, sensory-motor polyneuropathy; OH, orthostatic hypotension; PVC, premature ventricular contractions; PAC, premature atrial contractions*.

**Table 2:** Examination results of symptomatic ATTR patients with Glu61Lys mutation of symptomatic ATTRv patients with the Glu61Lys variant.

				Cardiac involvements				Clinical evaluation score				
Patient	Sural nerve biopsy	Biopsy of gastrocnemius muscle	Head-up tilt test	NT-ProBNP (pg/ml) (NV:< 125 pg/ml)	IVS (mm) (NV: ≤ 11 mm)	LVPW (mm) (NV:≤ 11 mm)	LVEF(%) (NV: ≥ 50%)	Cardiac magnetic resonance imaging	^99m^Tc-PYP	NIS	Norfolk QOL score	COMPASS-31 score
1	Moderately reduced fiber density, with small and large myelinated fibers.	Neuropathic pattern acccompanied with possible myopathic impairment, positive Congo-red and TTRstaining	Positive	160	15	13	65.8	Positive	Positive	74.5	56	24.50
2	Moderately reduced fiber density, predominant with small and large myelinated fibers.	Neuropathic pattern accompanied with possible myopathic impairment, positive Congo-red and TTR staining	Negative	2,234	13	13	54.9	Positive	Positive	60	56	25.35
3	ND	ND	Negative	78	12	11	42	ND	ND	10	11	7.80
4	ND	ND	Positive	74	12	12	65	Positive	Positive	81	48	27.12
5	ND	ND	Negative	52	20	17	54.7	ND	ND	54	80	10.00

*Abbreviations: COMPASS-31, Composite Autonomic Symptom Score 31; IVS, interventricular septal; LVEF, left ventricular ejection fraction; LVPW, left ventricular posterior wall; ND, not done; Norfolk QOL score, Norfolk Quality of life-diabetic neuropathy score; NIS, neuropathy impairment score; PYP, ^99m^ Tc-pyrophosphate*.

### Electrophysiology

All patients had severely reduced amplitudes of the CMAPs of the tibial nerves. Patient 1, 2, and 3 had prolonged DMLs of the tibial nerves. Patient 2, 3, 4, and 5 had mildly slowed MCV of the tibial nerves. Patient 1, 2, and 4 had severely reduced amplitudes of the CMAPs of the peroneal nerves, and for patient 5 the amplitude of the CMAPs of the peroneal nerve was not elicited. Patient 1, 2, and 4 had mildly prolonged DMLs of the peroneal nerves, and patient 1, 3, and 4 had mildly slowed MCV of the peroneal nerves. Patient 2, 4, and 5 had moderately reduced amplitudes of the CMAPs of the median nerves, and patient 1, 2, 3, and 4 had prolonged DMLs of the median nerves. Patient 1, 3, and 4 had mildly prolonged DMLs of the ulnar nerves, and patient 1 and 2 had mildly slowed MCV of the ulnar nerves. For the sensory nerve conduction study, the SNAPs of the median nerves for four patients were not elicited except for patient 3, and the four patients had varying degrees of abnormalities in the ulnar nerves. The superficial peroneal nerves and the sural nerves of all patients were severely impaired (Table 3).

**Table 3:** Neuro-electrophysiological features of symptomatic ATTRv patients with the Glu61Lys variant.

Nerves	Parameters	1	2	3	4	5	Normal values
Median motor nerves	DML, ms	**4.09**	**4.31**	**5.08**	**4.69**	3.85	<4
	CMAP, mV	5	**4.9**	5.8	**3.26**	**3.9**	>5
	MCV, m/s	51.1	53.4	51.6	**48.9**	**48.9**	>50
Ulnar motor nerves	DML, ms	**3.63**	2.62	**3.31**	**3.28**	2.31	<3
	CMAP, mV	8.4	8.7	7.2	6.67	5.8	>4
	MCV, m/s	**46.2**	**46.1**	57.9	57.6	51.0	>50
Peroneal motor nerves	DML, ms	**5.38**	**5.73**	4.54	**5.52**	**NR**	<5.3
	CMAP, mV	**0.24**	**0.19**	3.1	**0.42**	**NR**	>2
	MCV, m/s	**30.3**	44.7	**36.6**	**37.3**	**NR**	>40
Tibial motor nerves	DML, ms	**7.34**	**7.78**	**6.71**	4.9	3.43	<5
	CMAP, mV	**0.22**	**0.06**	**0.86**	**0.11**	**0.28**	>3.5
	MCV, m/s	**NR**	**30.0**	**39.3**	**40.0**	**33.8**	>40
Median sensory nerves	SNAP, μV	**NR**	**NR**	**4.0**	**NR**	**NR**	>5
	SCV, m/s	**NR**	**NR**	**45.0**	**NR**	**NR**	>50
Ulnar sensory nerves	SNAP, μV	4.3	**2.3**	17.3	**NR**	**NR**	>3
	SCV, m/s	**41.1**	**35.7**	63.7	**NR**	**NR**	>50
Superficial peroneal nerves	SNAP, μV	**NR**	**NR**	**NR**	**NR**	**NR**	>1
	SCV, m/s	**NR**	**NR**	**NR**	**NR**	**NR**	>40
Sural nerves	SNAP, μV	**NR**	**NR**	**NR**	**NR**	**NR**	>1
	SCV, m/s	**NR**	**NR**	**NR**	**NR**	**NR**	>40

*Abbreviations: CMAP, compound muscle action potential; DML, distal motor latency; MCV, motor conduction velocity; NR, no response; SCV, sensory conduction velocity; SNAP, sensory nerve action potential. The CMAPs were measured from peak to base. The abnormal values were printed in bold*.

### Pathology

Both patient 1 and 2 underwent nerve and muscle biopsies. The sural nerve pathology revealed severely reduced myelinated nerve fiber densities from the semithin section in patient 1 and 2 (Figure 1C). Axonal degeneration was observed in patient 2. There were some thickening basement membranes of some capillaries in patient 1. There were no onion bulbs of myelinated fibers observed. In both cases, there were no positive materials found by Congo-red staining and TTR immunohistochemical staining in sural nerves.

The biopsies of gastrocnemius muscle were also performed in patient 1 and 2. HE staining showed atrophic angular and round muscle fibers in both patients, and some necrotic muscle fibers with inflammatory cell infiltration, and some muscle fibers with rimmed vacuoles in patient 1 and 2 (Figure 1D). The fibers of angular and round atrophy were distributed in groups, involving two types, which were seen in adenosine triphosphatase staining and typical target-like muscle fibers with nicotinamide adenine dinucleotide tetrazolium reductase staining were found in patient 2 (Figure 1E). In both patients, the amyloid deposits in perimysium and perivascular areas were found by Congo-red staining and showed apple-green birefringence viewed under the polarized light, containing TTR deposits by immunohistochemical staining (Figures 1F–H).

### Thermodynamic and kinetic stability of E61K-TTR mutant protein

**Figure 2:**
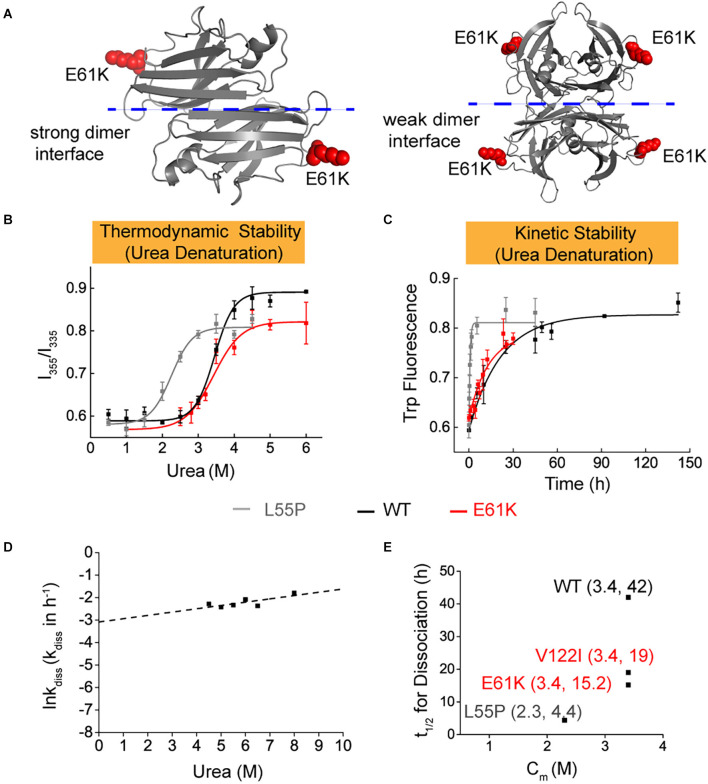
Thermodynamic and kinetic stability of E61K-TTR mutant protein. **(A)** X-ray crystallographic structure of E61K-TTR, modified based on previously reported structure (PDB code: 1BMZ). K61 was highlighted on the DE loop in red. **(B)** Thermodynamic stability of TTR and its mutants was measured by urea denaturation curve using tryptophan intrinsic fluorescence. **(C)** Kinetic stability of TTR and its mutants was measured by tryptophan fluorescence in 6.5 M urea. L55P, gray curve; WT, black curve; E61K, red curve. **(D)** The logarithm of the rate of tetramer dissociation, lnkdiss (kdiss in h-1), was plotted as a function of urea concentrations. The lnkdiss vs. urea concentration plot is linear, allowing extrapolation to 0 M urea. **(E)** Summary of thermodynamic (X-axis; Cm values) and kinetic (Y-axis; *t*_1/2_ of tetramer dissociation) stability, indicating its compromised kinetic stability in E61K-TTR proteins.

To better explain the clinical observations and understand the pathogenicity of E61K patients and asymptomatic carriers, efforts were made to characterize the thermodynamic and kinetic stabilities of E61K-TTR (Figure 2A) together with WT-TTR and a commonly studied L55P-TTR mutant as controls for direct comparison. A series of biochemical experiments showed that E61K-TTR exhibited comparable thermodynamic stability (Cm = 3.4 M) to WT-TTR (Cm = 3.4 M; Figures 2B,E). Nevertheless, E61K-TTR (*t*_1/2_ = 15.2 h) was kinetically destabilized in comparison to WT-TTR (*t*_1/2_ = 42 h), but more stable than that of L55P (*t*_1/2_ = 4.4 h; Figures 2C–E). These results indicated that the E61K-TTR mutant protein showed compromised kinetic stability by lowering the energy barrier of the rate-limiting tetramer dissociation step.

**Figure 3:**
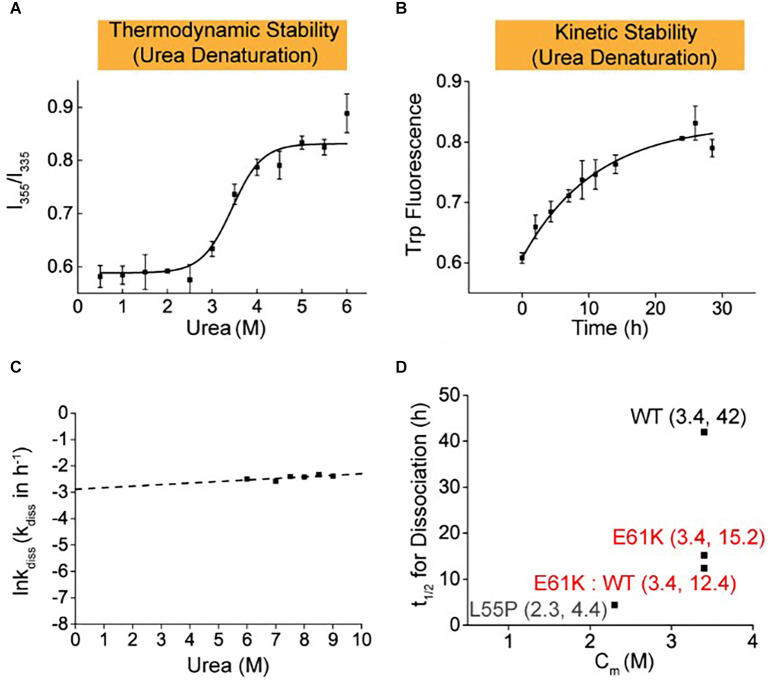
Thermodynamic and kinetic stability of heterozygous E61K:WT-TTR mixture proteins. Heterozygous protein mixture mimicking the scenario in patients’ plasma was prepared by mixing E61K-TTR and WT-TTR in equal amounts at 4°C for 48 h to allow for sufficient subunit exchange. **(A)** Thermodynamic stability of E61K-TTR after subunit exchange with WT-TTR. **(B)** Kinetic stability of E61K-TTR after subunit exchange with WT-TTR. **(C)** The logarithm of the rate of tetramer dissociation, lnkdiss (kdiss in h-1), was plotted as a function of urea concentrations. The lnkdiss vs. urea concentration plot is linear, allowing extrapolation to 0 M urea. **(D)** Summary of thermodynamic (X-axis; Cm values) and kinetic (Y-axis; *t*_1/2_ of tetramer dissociation) stability of heterozygous E61K:WT-TTR proteins. These results indicate E61K subunit can compromise the kinetic stability of WT, but WT subunit cannot stabilize E61K-TTR.

To better mimic the patients’ scenario with heterozygous TTR mutation in blood plasma, the thermodynamic and kinetic stability of the E61K:WT-TTR heterozygous tetramer protein was measured. WT- and E61K-TTR recombinant proteins were mixed for complete subunit exchange. Interestingly, it was observed that exchange with WT-TTR increased neither the thermodynamic stability (Cm E61K:WT = 3.4 M, Figures 3A,D) nor the kinetic stability (*t*_1/2_ E61K:WT = 12.4 h, Figures 3B–D) compared to its homozygous E61K-TTR proteins. In fact, the inclusion of WT-TTR in E61K-TTR mutant further compromised the kinetic stability from 15.2 to 12.4 h, suggesting subtle conformational changes upon WT-TTR exchange with E61K-TTR.

### E61K:WT-TTR heterozygous mutant protein responds poorly to kinetic stabilizers

Many small molecule kinetic stabilizers have been reported to inhibit TTR-related amyloidosis and some have been approved for clinical applications or clinical trials, such as Tafamidis, Diflunisal, and AG10. Among them, Tafamidis has been approved in many countries for the treatment of patients with ATTRv amyloidosis by stabilizing its tetramer *via* binding to two sites of TTR and inhibiting amyloid fibril formation. In this work, the ability of Tafamidis, AG10, and Diflunisal to inhibit the amyloid formation of E61K-TTR homozygous proteins and E61K:WT-TTR heterozygous tetramers was also studied.

**Figure 4:**
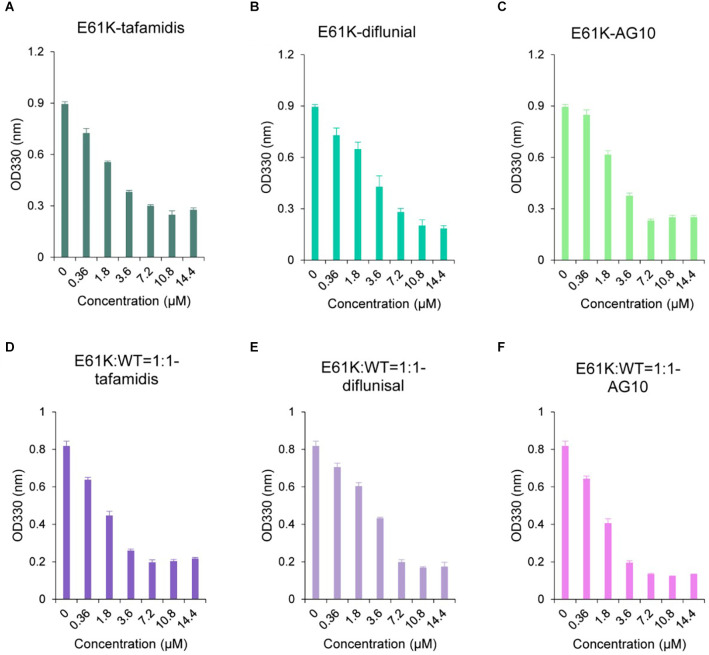
Comparison of the inhibitory effect of small molecule stabilizers on fiber formation of pure E61K-TTR and E61K:WT-TTR heterozygous mixture. **(A)** Tafamidis with E61K-TTR. **(B)** Diflunisal with E61K-TTR. **(C)** AG10 with E61K-TTR. **(D)** Tafamidis with E61K:WT-TTR was measured after mixing E61K-TTR and WT-TTR in equal amounts at 4°C for 48 h. **(E)** Diflunisal with E61K:WT-TTR. **(F)** AG10 with E61K:WT-TTR.TTR concentration was 3.6 μM. Fibril formation was induced by incubating the TTR protein-small molecule complexes in acidic buffer (NaOAc 200 mM, KCl 100 mM, acidified by AcOH to pH = 4.4) at 37°C for 72 h. The amount of TTR fibrils was determined by measuring the turbidity at 330 nm. Heterozygous E61K:WT-TTR exhibited similarly poor responses towards small molecule stabilizers compared to its pure E61K-TTR proteins.

It was found that Tafamidis, Diflunisal, and AG10 could inhibit the fibril formation of E61K-TTR (Figures 4A–C). However, as the concentration of drugs increased, no dose-response was observed for any of these drugs, indicating the widely reported negative cooperativity of TTR mutants for the second binding sites. Similarly, the heterozygous E61K: WT-TTR proteins responded poorly to all kinetic stabilizers with a minimal difference from the E61K-TTR homozygous proteins. These results suggested that E61K patients might respond poorly to current kinetic stabilizers, this might be due to negative cooperativity, resulting in the inability of the drug to enter or only a small amount into the second T4 binding site. For example, AG10 was reported to enter the second T4 binding site with weaker binding affinity due to the negative cooperativity (Penchala et al., 2013). In addition, since drugs bind to different TTR mutants with different binding affinity, the ability to enter the second T4 binding site was also influenced by the type of mutant. Meanwhile, it also highlighted the significance of characterizing these biochemical properties prior to precision medical treatment for ATTRv amyloidosis patients carrying different TTR mutations.

### Quantification of tetrameric TTR concentration in serum of patients and asymptomatic carriers

**Figure 5:**
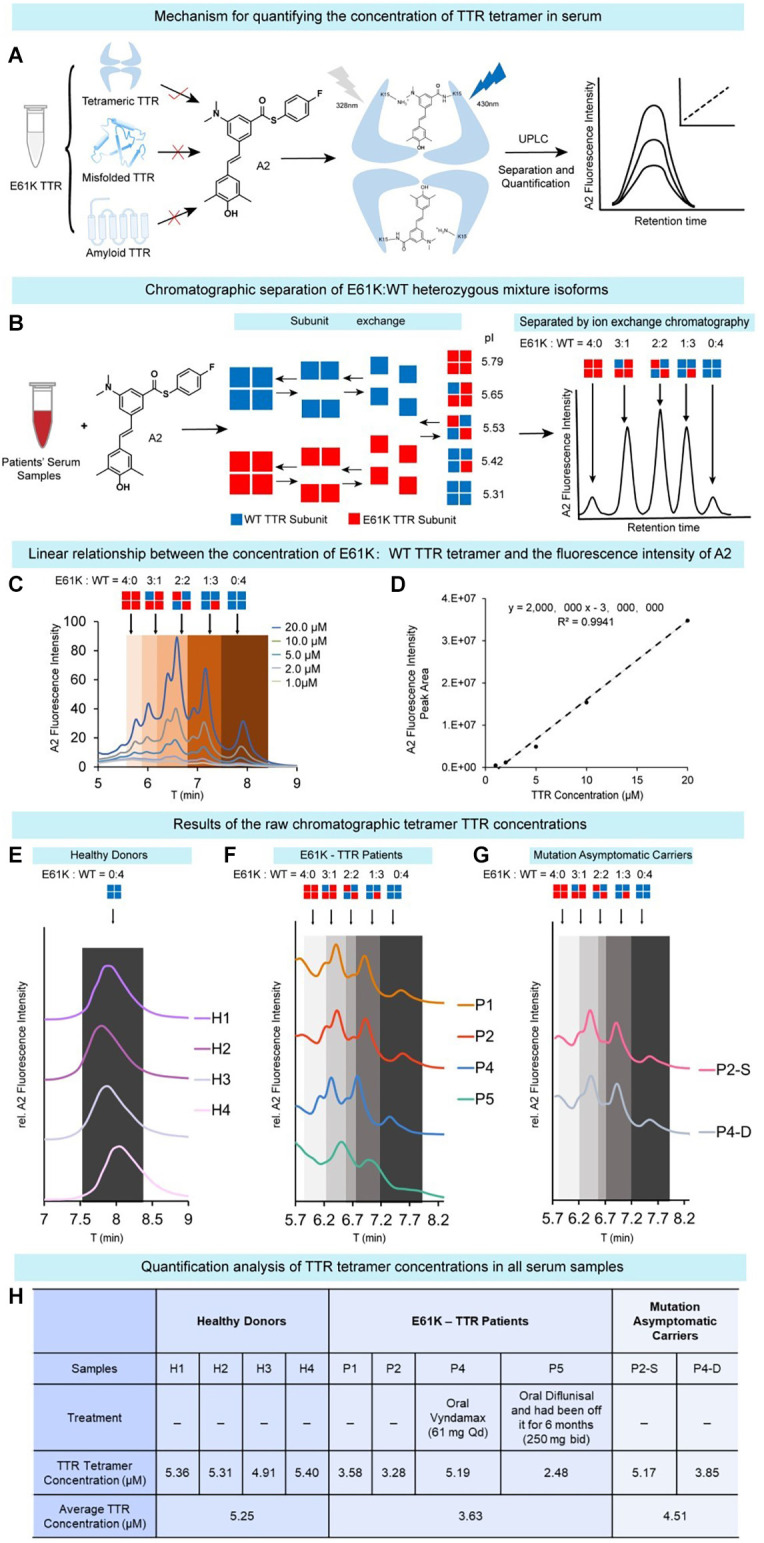
Quantification of tetrameric TTR concentration inblood serum samples of E61K mutation patients and carriers. **(A)** The working principle of chromatographic method to quantify tetrameric TTR in serum. The concentration of TTR tetramer in serum was quantified by selectively and exclusively labeling tetrameric TTR using a fluorescence turn-on probe A2 reported by the Kelly group. Using UPLC coupled with fluorescence detector, the concentration of tetrameric TTR can be quantified based on the fluorescence peak signals. **(B)** Chromatographic separation of E61K:WT heterozygous mixture isoforms. Taking advantage of E to K mutation, the pI values of these mixtures of different subunit ratios are different, leading to their separation on a ion-exchange chromatography. **(C,D)** Linear relationship between the concentration of recombinant E61K TTR tetramerlabelled by A2 probe and A2 fluorescence peak intensity. **(E–G)** The raw chromatographic tetramer TTR concentration results from four healthy donors **(E)**, four E61K-TTR patients **(F)**, and two mutation asymptomatic carriers **(G)** on UPLC detected by A2-probe-facilitated fluorescence chromatographic method. P2-S, P2’s son. P4-D, P4’s daughter. **(H)** Quantitative analysis of the TTR tetramer concentrations in all serum samples.

ATTRv amyloidosis is caused by the misfolding and aggregation of tetrameric mutant TTR proteins, and therefore the tetramer concentration fluctuations in blood serum may reflect the progressiveness of this disease (a decrease in tetramer concentration) and the response to kinetic stabilizer treatment (an increase in tetramer concentration). Due to the compromised kinetic stability in the above experimental results, there is reason to speculate that the serum concentration of TTR tetramers in E61K patients would be lower than that in healthy donors. To this end, serum samples were collected from four patients and two asymptomatic carriers of the E61K mutation as well as four wild type healthy donors in this study. In order to quantify the tetrameric TTR concentration in blood serum samples, fluorescence based ultra-performance liquid chromatography (UPLC) method reported by Kelly’s group (Rappley et al., 2014) was used to quantitatively measure the concentration of TTR tetramer in the serum samples (Figure 5A). Stilbene A2 probe selectively, covalently, and exclusively reacts with Lys-15 at the weak dimer-dimer interface of TTR tetramer and emits fluorescence at 430 nm. This probe does not label misfolded and amyloid TTR.

Meanwhile, as the patients’ TTR genes are heterozygous, both WT and mutant proteins are expressed. The serum of E61K mutation individuals contained both WT-TTR and E61K-TTR at the same time and their subunits tended to exchange in blood serum, resulting in five tetramer types of different subunit ratios (Figure 5B). When separated by UPLC ion-exchange chromatography, these tetramers of different isoelectric points (pI values) caused by their E to K mutation will result in different elution times (Figure 5B). Theoretically, five major peaks can be observed on the chromatogram (Figure 5B). In fact, the resulting chromatographic peak pattern is slightly more complex but can be roughly distinguished (Figures 5C,F,G). The complexity may arise from subtle conformational changes in serum and oxidations during sample preparation. Thus, for E61K:WT-TTR heterozygous individuals’ serum samples, not only can the tetramer concentration be quantified, but the different tetramer types consisting of different E61K:WT ratios be separated. The linear range of TTR concentration for the standard curve was determined as 0.5–10 μM (Figure 5D), well aligned within the human TTR concentration range.

Using this assay, the concentrations of TTR tetramer were measured in the serum samples of four wild type healthy donors (H1-H4), four symptomatic patients (P1, P2, P4, and P5), and two asymptomatic descendent carriers (P2-S, P4-D, Figures 5E–G). The concentration of tetrameric TTR was listed in Figure 5H. Overall, TTR tetramer concentration in healthy donors carrying wild-type TTR gene (5.25 μM) was higher than that in E61K ATTRv amyloidosis patients (3.63 μM, Figure 5H). On the other hand, TTR tetramer concentration in the asymptomatic E61K-TTR carriers (4.51 μM) was higher than that of symptomatic patients (3.63 μM, Figure 5H) but slightly lower than that of healthy donors, which was why the concentration of E61K-TTR tetramer in the serum could serve as a biomarker to monitor disease progression and therapeutic window time. A decrease in this parameter may alert clinicians to transition from asymptomatic carriers to symptomatic patients.

Among these patients, patient P4 took Vyndamax (Tafamidis) orally and patient P5 took Diflunisal orally, but patient P5 had been off the drug for 6 months. For these two patients on medication, P4 orally administrated with Vyndamax (Tafamidis) had elevated serum concentrations of TTR tetramers (5.19 μM), which was even higher than that of her asymptomatic daughter (3.85 μM) but equaled that of the asymptomatic P2’s son (5.17 μM). This observation was inconsistent with the *in-vitro* fibril formation inhibition experiment (Figure 4) possibly due to the differences in intake concentrations and pharmacokinetic and pharmacodynamic profiles between Tafamidis and Diflunisal. In contrast, for P5 off Diflunisal treatment (bid) for 6 months, the serum TTR tetramer concentration (2.48 μM) was much lower than that of P4 (5.19 μM). However, without the pre-Rx TTR levels from these patients, no conclusion regarding drug responses should be drawn based on these results.

## Discussion

TTR E61K mutation was reported for the first time by Shiomi et al. (1993) in Japan, and then five unrelated E61K families were consecutively reported between 2009 and 2018 in Asian populations (Noto et al., 2009; Murakami et al., 2017; Nakano et al., 2018). The E61K mutation was the third largest mutation on the TTR gene in our medical center except for V30M and A97S mutations. As was previously reported, all patients with E61K mutation in this study were late onset (He et al., 2019). The Chinese families we presented herein, had sensory-motor polyneuropathy, severe cardiomyopathy, and autonomic dysfunction as cardinal symptoms, without apparent evidence of ocular or renal involvement. Noto and Nakano et al. have reported severe cardiomyopathy in patients with E61K mutation (Shiomi et al., 1993; Murakami et al., 2017). Cardiac involvement was also common and could lead to symptoms of heart failure and pacemaker implantation in patients with A97S and V30M mutations (Hsu et al., 2017; Low et al., 2019; Ruberg et al., 2019; Rubin and Maurer, 2020). We were the first to report one patient with E61K mutation who presented with chronic dry cough, which was also observed in patients with V30M, A97S, F33V (p.Phe53Val), and E42G (p.Glu62Gly) mutations (Yuan et al., 2019; Du et al., 2021). In the previous report, one patient initially presented with symptoms of carpal tunnel syndrome (CTS; Noto et al., 2009; Table 1). Three families were from Shandong province of China and the other two families were from Hebei province and Jilin province of China respectively, all five families were from the north of China. The E61K mutation was mostly reported in Japan which is located in the northeast of China. We speculated that these patients might originate from common ancestors, while this speculation needed more researches to confirm.

Our cohort confirmed that all patients had predominantly sensory-motor axonal neuropathies in the electrophysiological studies. Electrophysiologically, the involvement of sensory nerves was more severe than that of motor nerves. and the involvement of lower limbs was more severe than that of upper limbs. For the sural nerve biopsy in our study, the loss of myelinated nerve fiber density was found. The loss of small myelinated nerve fibers, which were already present in asymptomatic carriers, was confirmed to be an initial event in ATTRv amyloidosis (Fernandes et al., 2019). For the gastrocnemius muscle biopsy, besides the neurogenic muscle fibers impairment, some muscle fibers with rimmed vacuoles were found, and the existence of TTR-related amyloid deposits was confirmed by Congo-red staining and TTR immunohistochemical staining. Patients with ATTRv amyloidosis had pathologically confirmed amyloid myopathy in previous reports (Liewluck and Milone, 2017), so we speculated that muscle impairment in ATTRv amyloidosis was not just secondary to neurogenic changes.

It was reported that the activation barrier associated with L55P-TTR tetramer dissociation was lower than the barrier for WT-TTR dissociation, and L55P-TTR was more likely to form transient conformations compatible with aggregation and amyloid formation in comparison with WT-TTR. Amongst TTR variants, the L55P mutation yielded early onset and aggressive symptoms with affected patients dying of peripheral nervous system, cardiac, and renal amyloidosis by age 35 (Jacobson et al., 1992; Lashuel et al., 1999; Rodrigues et al., 2010). In our study, E61K-TTR exhibited comparable thermodynamic stability to WT-TTR, and was kinetically destabilized in comparison to WT-TTR, but better than those early age-of-onset mutations (L55P, etc.). Clinically, E61K mutation was late onset and more benign compared to L55P mutation. Therefore, it was speculated that the kinetical and thermodynamic stabilities of various TTR mutations were partly consistent with clinical onset and severity.

It was observed in our study that heterozygous E61K-TTR proteins were more kinetically destabilized than homozygous E61K-TTR proteins. A prior study reported that homozygous V30M mutation did not implicate a more severe phenotype than heterozygous V30M mutation for Swedish patients (Holmgren et al., 2005), and Japanese researchers found that the presence of endogenous normal mouse TTR did not affect human variant TTR-derived amyloid deposition in models of V30M TTR transgenic mice (Kohno et al., 1997). However, it was reported from Japan and Spain that patients with homozygous V30M mutation had extremely early-onset, severe familial amyloid polyneuropathy and central nervous system symptoms, which might be due to a double dose of the mutant gene accelerating amyloid deposition on tissues (Munar-Qués et al., 2001; Yoshinaga et al., 2004; Tojo et al., 2008; Uchida et al., 2015). Another study about the stabilization of mutant human TTR in transgenic animals and murine TTR-knockouts expressing the L55P TTR transgene revealed that when a highly amyloidogenic human TTR was present in relatively low concentrations, the TTR tetramer was much more stable in the presence of the murine protein, for TTR was circulated as hybrid human/murine heterotetramers and the mouse protein might be inhibitory to aggregation and subsequent amyloid formation (Tagoe et al., 2007). Based on the above studies, it was assumed that stability varied between homozygous and heterozygous mutations in different TTR genotypes, but more research is needed to confirm this conclusion, for we currently have no clinical experience with homozygous E61K.

Data from large registry and referral center studies supported that Tafamidis, as a kinetic stabilizer, was associated with reductions in cardiovascular-related hospitalizations and conferring survival benefits in patients with stage 1 ATTR-PN (Maurer et al., 2018; Lamb and Deeks, 2019). Diflunisal acted as a tetramer stabilizer for the treatment of ATTR-CM, as the associated survival benefits were reported to be similar to those of Tafamidis (Maurer et al., 2018; Ibrahim et al., 2022). AG10 stabilized TTR tetramer by forming H-bonds with S117 (Zhou et al., 2020), and AG10 treatment was well-tolerated, achieved target plasma concentrations, and demonstrated near-complete stabilization of TTR (Judge et al., 2019; Nelson et al., 2021). In this work, it was found that E61K-TTR homozygous proteins and E61K: WT-TTR heterozygous tetramers might respond to current kinetic stabilizers less effectively compared to WT-TTR because full inhibition of fibril formation could be achieved for WT-TTR based on previously reported *in vitro* results. We speculated that maintaining high drug concentrations in blood plasma might have an effect on clinical efficacy.

There is much evidence that fibril formation proceeded from a misfolded TTR monomer generated from monomeric TTR subunits that were in equilibrium with the native TTR tetramer (Tagoe et al., 2007). In our study, TTR tetramer concentration, different from total serum TTR concentration, in healthy donors was higher than that in E61K ATTRv amyloidosis patients, in previously reported serum TTR concentrations in the controls were statistically significantly higher than in the TTR V30M carriers in Swedish patients, which were in the same range as those in African-Americans carrying the TTR V122I allele (Buxbaum et al., 2010). On the other hand, TTR tetramer concentration in the asymptomatic E61K-TTR carriers was higher than that of symptomatic patients but still lower than the reported range in healthy adult populations. These results indicated that the tetramer concentration reductions had occurred among some of asymptomatic carriers. TTR tetramer concentration in patients with Tafamidis was higher than that in E61K-TTR carriers, suggesting that Tafamidis could increase the TTR tetramer concentration, and even bring the TTR tetramer concentration close to the normal level. These results reminded us that TTR tetramer concentrations, as well as clinical symptoms and abnormalities of other indicators needed to be closely monitored. Decreases in TTR tetramer concentrations might call for attention to transition from asymptomatic carriers to symptomatic patients. However, the relationship between the decrease of TTR tetramer concentration and the amyloid formation nunclear, so more studies are needed.

## Conclusion

The patients with E61K mutation tended to be late-onset and showed sensory-motor polyneuropathy and severe cardiomyopathy as the main symptoms. The concentration of TTR tetramer in the serum might serve as a biomarker to monitor disease progress, therapeutic window time, and therapeutic response to TTR kinetic stabilizer drugs.

## Data Availability Statement

The raw data supporting the conclusions of this article will be made available by the authors, without undue reservation.

## Ethics Statement

The studies involving human participants were reviewed and approved by Human Research Ethics Committee of Peking University First Hospital. The patients/participants provided their written informed consent to participate in this study.

## Author Contributions

XC, LM, ZW, YY, and YL contributed to the conception and design of the study. XC and MW collected and organized the database and wrote the first draft of the manuscript. YH, ZW, WM, and RT wrote sections of the manuscript. JY, YZ, WS, YW, ZX, JD, and YC participated in the design. All authors contributed to the article and approved the submitted version.

## Funding

This study was supported by the National Natural Science Foundation of China under Grant number 82101469, 21907091; Beijing Municipal Natural Science Foundation under Grant number 7194323; and Peking University First Hospital Cross Clinical Research Project.

## Conflict of Interest

The authors declare that the research was conducted in the absence of any commercial or financial relationships that could be construed as a potential conflict of interest.

## Publisher’s Note

All claims expressed in this article are solely those of the authors and do not necessarily represent those of their affiliated organizations, or those of the publisher, the editors and the reviewers. Any product that may be evaluated in this article, or claim that may be made by its manufacturer, is not guaranteed or endorsed by the publisher.

## Abbreviations

ATTRv amyloidosis: hereditary transthyretin amyloidosis
ATTRv-CM: hereditary transthyretin amyloid cardiomyopathy
ATTRv-PN: hereditary transthyretin amyloid polyneuropathy
A97S: Ala97Ser
CMAPs: compound muscle action potentials
COMPASS-31: Composite Autonomic Symptom Score 31
CTS: carpal tunnel syndrome
DMLs: distal motor latencies
E61K: Glu61Lys
F33V: Phe33Val
HE: hematoxylin and eosin
L55P: Leu55Pro
L55P: motor conduction velocities
NIS: Neuropathy Impairment Score
Norfolk QOL-DN: Norfolk Quality of Life-Diabetic Neuropathy
SCVs: sensory conduction velocities
SNAPs: sensory nerve action potentials
99mTc-PYP: 99mTechnetium-pyrophosphate
TTR: transthyretin
UPLC: ultra-performance liquid chromatography
V30M: Val30Met
WT: Wild-type.

## References

[B1] Buxbaum J. Anan I. Suhr O. (2010). Serum transthyretin levels in Swedish TTR V30M carriers. Amyloid 17, 83–85. 10.3109/13506129.2010.483118 20462367

[B2] Buxbaum J. Jacobson Daniel R. Tagoe C. Alexander A. Kitzman D. W. Greenberg B.. (2006). Transthyretin V122I in African Americans with congestive heart failure. J. Am. Coll. Cardiol. 47, 1724–1725. 10.1016/j.jacc.2006.01.042 16631014

[B3] Conceição I. De Carvalho M. (2007). Clinical variability in type I familial amyloid polyneuropathy (Val30Met): comparison between late- and early-onset cases in Portugal. Muscle Nerve 35, 116–118. 10.1002/mus.20644 16969832

[B4] Du K. Li F. Wang H. Miao Y. Lv H. Zhang W.. (2021). Hereditary transthyretin amyloidosis in mainland China: a unicentric retrospective study. Ann. Clin. Transl. Neurol. 8, 831–841. 10.1002/acn3.51328 33739616 PMC8045954

[B5] Fernandes A. Coelho T. Rodrigues A. Felgueiras H. Oliveira P. Guimarães A.. (2019). Clinicopathological correlations of sural nerve biopsies in TTR Val30Met familial amyloid polyneuropathy. Brain Commun. 1:fcz032. 10.1093/braincomms/fcz032 32954271 PMC7425381

[B6] Hammarström P. Jiang X. Hurshman A. R. Powers E. T. Kelly J. W. (2002). Sequence-dependent denaturation energetics: a major determinant in amyloid disease diversity. Proc. Natl. Acad. Sci. U S A 99, 16427–16432. 10.1073/pnas.202495199 12351683 PMC139904

[B7] He S. Tian Z. Guan H. Li J. Fang Q. Zhang S. (2019). Clinical characteristics and prognosis of Chinese patients with hereditary transthyretin amyloid cardiomyopathy. Orphanet J. Rare Dis. 14:251. 10.1186/s13023-019-1235-x 31718691 PMC6852775

[B8] Holmgren G. Hellman U. Lundgren H. E. Sandgren O. Suhr O. B. (2005). Impact of homozygosity for an amyloidogenic transthyretin mutation on phenotype and long term outcome. J. Med. Genet. 42, 953–956. 10.1136/jmg.2005.033720 15930086 PMC1735971

[B9] Hsu H.-C. Liao M. F. Hsu J. L. Lo A. L. Kuo H. C. Lyu R. K.. (2017). Phenotypic expressions of hereditary Transthyretin Ala97Ser related Amyloidosis (ATTR) in Taiwanese. BMC Neurol. 17:178. 10.1186/s12883-017-0957-4 28882124 PMC5590125

[B10] Ibrahim M. Saint Croix G. R. Lacy S. Fattouh M. Barillas-Lara M. I. Behrooz L.. (2022). The use of diflunisal for transthyretin cardiac amyloidosis: a review. Heart Fail. Rev. 27, 517–524. 10.1007/s10741-021-10143-4 34272629

[B11] Jacobson D. R. McFarlin D. E. Kane I. Buxbaum J. N. (1992). Transthyretin Pro55, a variant associated with early-onset, aggressive, diffuse amyloidosis with cardiac and neurologic involvement. Hum. Genet. 89, 353–356. 10.1007/BF00220559 1351039

[B12] Judge D. P. Heitner S. B. Falk R. H. Maurer M. S. Shah S. J. Witteles R. M.. (2019). Transthyretin stabilization by AG10 in symptomatic transthyretin amyloid cardiomyopathy. J. Am. Coll. Cardiol. 74, 285–295. 10.1016/j.jacc.2019.03.012 30885685

[B13] Kanda Y. Goodman D. S. Canfield R. E. Morgan F. J. (1974). The amino acid sequence of human plasma prealbumin. J. Biol. Chem. 249, 6796–6805. 10.1016/S0021-9258(19)42128-5 4607556

[B14] Kohno K. Palha J. A. Miyakawa K. Saraiva M. J. Ito S. Mabuchi T.. (1997). Analysis of amyloid deposition in a transgenic mouse model of homozygous familial amyloidotic polyneuropathy. Am. J. Pathol. 150, 1497–1508. 10.1081/MA-120017258 9095004 PMC1858187

[B15] Koike H. Misu K. Sugiura M. Iijima M. Mori K. Yamamoto M.. (2004). Pathology of early- vs. late-onset TTR Met30 familial amyloid polyneuropathy. Neurology 63, 129–138. 10.1212/01.wnl.0000132966.36437.12 15249622

[B16] Koike H. Tanaka F. Hashimoto R. Tomita M. Kawagashira Y. Iijima M.. (2012). Natural history of transthyretin Val30Met familial amyloid polyneuropathy: analysis of late-onset cases from non-endemic areas. J. Neurol. Neurosurg. Psychiatry 83, 152–158. 10.1136/jnnp-2011-301299 22228785

[B17] Lamb Y. N. Deeks E. D. (2019). Tafamidis: a review in transthyretin amyloidosis with polyneuropathy. Drugs 79, 863–874. 10.1007/s40265-019-01129-6 31098895

[B18] Lashuel H. A. Wurth C. Woo L. Kelly J. W. (1999). The most pathogenic transthyretin variant, L55P, forms amyloid fibrils under acidic conditions and protofilaments under physiological conditions. Biochemistry 38, 13560–13573. 10.1021/bi991021c 10521263

[B20] Liewluck T. Milone M. (2017). Characterization of isolated amyloid myopathy. Eur. J. Neurol. 24, 1437–1445. 10.1111/ene.13448 28888072

[B21] Low S. C. Tan C. Y. Md Sari N. A. Ahmad-Annuar A. Wong K. T. Lin K. P.. (2019). Ala97Ser mutation is common among ethnic Chinese Malaysians with transthyretin familial amyloid polyneuropathy. Amyloid 26, 7–8. 10.1080/13506129.2019.1582479 31343308

[B23] Maurer M. S. Schwartz J. H. Gundapaneni B. Elliott P. M. Merlini G. Waddington-Cruz M.. (2018). Tafamidis treatment for patients with transthyretin amyloid cardiomyopathy. N. Engl. J. Med. 379, 1007–1016. 10.1056/NEJMoa1805689 30145929

[B24] Monaco H. L. Rizzi M. Coda A. (1995). Structure of a complex of two plasma proteins: transthyretin and retinol-binding protein. Science 268, 1039–1041. 10.1126/science.7754382 7754382

[B25] Munar-Qués M. López Domínguez J. M. Viader-Farré C. Moreira P. Saraiva M. J. (2001). Two Spanish sibs with familial amyloidotic polyneuropathy homozygous for the V30M-TTR gene. Amyloid 8, 121–123. 10.3109/13506120109007355 11409034

[B26] Murakami T. Nishimura H. Nagai T. Hemmi S. Kutoku Y. Ohsawa Y.. (2017). Clinical and pathological findings in familial amyloid polyneuropathy caused by a transthyretin E61K mutation. J. Neurol. Sci. 381, 55–58. 10.1016/j.jns.2017.08.017 28991715

[B27] Murakami T. Ohsawa Y. Zhenghua L. Yamamura K. Sunada Y. (2010). The transthyretin gene is expressed in Schwann cells of peripheral nerves. Brain Res. 1348, 222–225. 10.1016/j.brainres.2010.06.017 20547140

[B28] Murakami T. Yokoyama T. Mizuguchi M. Toné S. Takaku S. Sango K.. (2021). A low amyloidogenic E61K transthyretin mutation may cause familial amyloid polyneuropathy. J. Neurochem. 156, 957–966. 10.1111/jnc.15162 32852783

[B29] Nakano Y. Tadokoro K. Ohta Y. Sato K. Takemoto M. Hishikawa N.. (2018). Two cases of late onset familial amyloid polyneuropathy with a Glu61Lys transthyretin variant. J. Neurol. Sci. 390, 22–25. 10.1016/j.jns.2018.04.003 29801893

[B30] Nelson L. T. Paxman R. J. Xu J. Webb B. Powers E. T. Kelly J. W. (2021). Blinded potency comparison of transthyretin kinetic stabilisers by subunit exchange in human plasma. Amyloid 28, 24–29. 10.1080/13506129.2020.1808783 32811187 PMC7952025

[B31] Noto Y. Tokuda T. Shiga K. Tsuchiya A. Yazaki M. Matoba S.. (2009). Cardiomyopathy in a Japanese family with the Glu61Lys transthyretin variant: a new phenotype. Amyloid 16, 99–102. 10.1080/13506120902879335 20536403

[B32] Penchala S. C. Connelly S. Wang Y. Park M. S. Zhao L. Baranczak A.. (2013). AG10 inhibits amyloidogenesis and cellular toxicity of the familial amyloid cardiomyopathy-associated V122I transthyretin. Proc. Natl. Acad. Sci. U S A 110, 9992–9997. 10.1073/pnas.1300761110 23716704 PMC3683741

[B33] Rappley I. Monteiro C. Novais M. Baranczak A. Solis G. Wiseman R. L.. (2014). Quantification of transthyretin kinetic stability in human plasma using subunit exchange. Biochemistry 53, 1993–2006. 10.1021/bi500171j 24661308 PMC3977577

[B34] Raz A. Goodman D. S. (1969). The interaction of thyroxine with human plasma prealbumin and with the prealbumin-retinol-binding protein complex. J. Biol. Chem. 244, 3230–3237. 4978316

[B35] Refai E. Dekki N. Yang S. N. Imreh G. Cabrera O. Yu L.. (2005). Transthyretin constitutes a functional component in pancreatic β-cell stimulus-secretion coupling. Proc. Natl. Acad. Sci. U S A 102, 17020–17025. 10.1073/pnas.0503219102 16286652 PMC1287967

[B36] Robinson L. Z. Reixach N. (2014). Quantification of quaternary structure stability in aggregation-prone proteins under physiological conditions: the transthyretin case. Biochemistry 53, 6496–6510. 10.1021/bi500739q 25245430 PMC4204887

[B37] Rodrigues J. R. Simões C. J. Silva C. G. Brito R. M. (2010). Potentially amyloidogenic conformational intermediates populate the unfolding landscape of transthyretin: insights from molecular dynamics simulations. Protein Sci. 19, 202–219. 10.1002/pro.289 19937650 PMC2865717

[B38] Ruberg F. L. Grogan M. Hanna M. Kelly J. W. Maurer M. S. (2019). Transthyretin amyloid cardiomyopathy: JACC state-of-the-art review. J. Am. Coll. Cardiol. 73, 2872–2891. 10.1016/j.jacc.2019.04.003 31171094 PMC6724183

[B39] Rubin J. Maurer M. S. (2020). Cardiac amyloidosis: overlooked, underappreciated and treatable. Annu. Rev. Med. 71, 203–219. 10.1146/annurev-med-052918-020140 31986086

[B40] Shiomi K. Nakazato M. Matsukura S. Ohnishi A. Hatanaka H. Tsuji S.. (1993). A basic transthyretin variant (Glu61–>Lys) causes familial amyloidotic polyneuropathy: protein and DNA sequencing and PCR-induced mutation restriction analysis. Biochem. Biophys. Res. Commun. 194, 1090–1096. 10.1006/bbrc.1993.1933 8352764

[B41] Soprano D. R. Herbert J. Soprano K. J. Schon E. A. Goodman D. S. (1985). Demonstration of transthyretin mRNA in the brain and other extrahepatic tissues in the rat. J. Biol. Chem. 260, 11793–11798. 10.1016/S0021-9258(17)39100-7 4044580

[B42] Stauder A. J. Dickson P. W. Aldred A. R. Schreiber G. Mendelsohn F. A. Hudson P. (1986). Synthesis of transthyretin (pre-albumin) mRNA in choroid plexus epithelial cells, localized by in situ hybridization in rat brain. J. Histochem. Cytochem. 34, 949–952. 10.1177/34.7.3458812 3458812

[B43] Tagoe C. E. Reixach N. Friske L. Mustra D. French D. Gallo G.. (2007). *In vivo* stabilization of mutant human transthyretin in transgenic mice. Amyloid 14, 227–236. 10.1080/13506120701464396 17701470

[B44] Tojo K. Sekijima Y. Machida K. Tsuchiya A. Yazaki M. Ikeda S. (2008). Amyloidogenic transthyretin Val30Met homozygote showing unusually early-onset familial amyloid polyneuropathy. Muscle Nerve 37, 796–803. 10.1002/mus.21028 18506713

[B45] Uchida Y. Takada K. Tsugu Y. Ueda M. Yamashita T. Ando Y.. (2015). Two brothers homozygous for the TTR V30M both presenting with a phenotype dominated by central nervous complications. Amyloid 22, 261–262. 10.3109/13506129.2015.1104661 26587769

[B46] Wang S. Peng W. Pang M. Mao L. Peng D. Yu B.. (2022). Clinical profile and prognosis of hereditary transthyretin amyloid cardiomyopathy: a single-center study in south China. Front. Cardiovasc. Med. 9:900313. 10.3389/fcvm.2022.900313 35833187 PMC9271707

[B47] Yoshinaga T. Takei Y.-i Katayanagi K. Ikeda S.-i (2004). Postmortem findings in a familial amyloid polyneuropathy patient with homozygosity of the mutant Val30Met transthyretin gene. Amyloid 11, 56–60. 10.1080/13506120410001688581 15185500

[B48] Yuan Z. Guo L. Liu X. Xiao X. Jiao B. Wang J.. (2019). Familial amyloid polyneuropathy with chronic paroxysmal dry cough in Mainland China: a Chinese family with a proven heterozygous missense mutation c.349G>T in the transthyretin gene. J. Clin. Neurosci. 60, 164–166. 10.1016/j.jocn.2018.10.040 30361054

[B49] Zhou S. Ge S. Zhang W. Zhang Q. Yuan S. Lo G. V.. (2020). Conventional molecular dynamics and metadynamics simulation studies of the binding and unbinding mechanism of TTR stabilizers AG10 and tafamidis. ACS Chem. Neurosci. 11, 3025–3035. 10.1021/acschemneuro.0c00338 32915538

